# The New Seafloor Observatory (OBSEA) for Remote and Long-Term Coastal Ecosystem Monitoring

**DOI:** 10.3390/s110605850

**Published:** 2011-05-31

**Authors:** Jacopo Aguzzi, Antoni Mànuel, Fernando Condal, Jorge Guillén, Marc Nogueras, Joaquin del Rio, Corrado Costa, Paolo Menesatti, Pere Puig, Francesc Sardà, Daniel Toma, Albert Palanques

**Affiliations:** 1 Instituto de Ciencias del Mar (ICM-CSIC), Paseo Maritimo de la Barceloneta, 37-49, 08003 Barcelona, Spain; E-Mails: fernandocondal@gmail.com (F.C.); jorge@icm.csic.es (J.G.); ppuig@icm.csic.es (P.P.); siscu@icm.csic.es (F.S.); albertp@icm.csic.es (A.P.); 2 SARTI Research Group, Electronics Department, Universitat Politècnica de Catalunya (UPC), Rambla de la Exposición 24, 08800 Vilanova i la Geltrú-Barcelona, Spain; E-Mails: antoni.manuel@upc.edu (A.M.); marc.nogueras@upc.edu (M.N.); joaquin.del.rio@upc.edu (J.D.R.); daniel.mihai.toma@upc.edu (D.T.); 3 Agritechlab, Agricultural Engineering Research Unit, Agriculture Research Council (CRA-ING), Via della Pascolare, 16, 00015 Monterotondo Scalo-Rome, Italy; E-Mails: corrado.costa@entecra.it (C.C.); paolo.menesatti@entecra.it (P.M.)

**Keywords:** OBSEA, cabled observatories, multidisciplinary observation, EMSO ESONET, remote ecosystem monitoring, automated video image analysis, fish community, activity rhythms

## Abstract

A suitable sampling technology to identify species and to estimate population dynamics based on individual counts at different temporal levels in relation to habitat variations is increasingly important for fishery management and biodiversity studies. In the past two decades, as interest in exploring the oceans for valuable resources and in protecting these resources from overexploitation have grown, the number of cabled (permanent) submarine multiparametric platforms with video stations has increased. Prior to the development of seafloor observatories, the majority of autonomous stations were battery powered and stored data locally. The recently installed low-cost, multiparametric, expandable, cabled coastal Seafloor Observatory (OBSEA), located 4 km off of Vilanova i la Gertrú, Barcelona, at a depth of 20 m, is directly connected to a ground station by a telecommunication cable; thus, it is not affected by the limitations associated with previous observation technologies. OBSEA is part of the European Multidisciplinary Seafloor Observatory (EMSO) infrastructure, and its activities are included among the Network of Excellence of the European Seas Observatory NETwork (ESONET). OBSEA enables remote, long-term, and continuous surveys of the local ecosystem by acquiring synchronous multiparametric habitat data and bio-data with the following sensors: Conductivity-Temperature-Depth (CTD) sensors for salinity, temperature, and pressure; Acoustic Doppler Current Profilers (ADCP) for current speed and direction, including a turbidity meter and a fluorometer (for the determination of chlorophyll concentration); a hydrophone; a seismometer; and finally, a video camera for automated image analysis in relation to species classification and tracking. Images can be monitored in real time, and all data can be stored for future studies. In this article, the various components of OBSEA are described, including its hardware (the sensors and the network of marine and land nodes), software (data acquisition, transmission, processing, and storage), and multiparametric measurement (habitat and bio-data time series) capabilities. A one-month multiparametric survey of habitat parameters was conducted during 2009 and 2010 to demonstrate these functions. An automated video image analysis protocol was also developed for fish counting in the water column, a method that can be used with cabled coastal observatories working with still images. Finally, bio-data time series were coupled with data from other oceanographic sensors to demonstrate the utility of OBSEA in studies of ecosystem dynamics.

## 1. Introduction

Limitations in sampling repeatability can produce spatial and temporal biases in stock and biodiversity assessment based on rhythmic and stochastic responses of species behaviour [1]. Traditional sampling tools include methods such as visual surveys by divers in shallow coastal areas [2–4] and trawling on shelves and slopes [5,6]. Sampling outcomes from these methods are affected by two temporal dynamics: the dynamics of contingent environmental events such as atmospheric-driven perturbations (*i.e.*, species punctual response [7,8]) and the dynamics of deterministic fluctuations in habitat parameters (*i.e.*, geophysical cycles) such as light intensity and tidally driven water motions (*i.e.*, species rhythmic response [9,10]). Marine species display rhythmic behaviours in response to geophysical cycles, and these responses affect observable populations during field sampling [11]. The time of day and the season can impact sampling planning if they are not considered as conditioning variables [12].

The available data on the presence and abundance of marine species are often too widely separated in both space and time to allow for a reliable evaluation of population demography and local biodiversity, mainly due to technological limitations in observational capability [11]. These limitations refer not only to animal detection but also to our capabilities for gathering contextual geological, chemical, and physical oceanographic information around the biological record, which in turn constrains our understanding of the underlying habitat regulation process. Both the punctual and rhythmic responses of species to environmental changes require continuous observations to produce synchronous time series of habitat and bio-data and to accurately model ecosystem functioning [13]. Multiparametric platforms are an interesting progression in this context. All platforms meet the two major needs of present marine research: continuity and duration in data collection as well as the remote and real-time supervision of ecological processes. Cabled observatories are, in fact, planned to continuously measure several habitat parameters that affect ecosystem functioning [14].

In the past decades, the number of cabled observatories has increased in both coastal and deep-sea areas [15–18]. Several countries have started programs of long-term multiparametric monitoring centred on the installation of cabled observatories at different depths at the continental margins [16,18,19]. Their purposes are markedly different, including monitoring earthquake [20] and capturing Earth-crossing neutrinos [21]. When bearing video cameras, important bio-data time series can be also obtained in relation to the presence of species and their populations [22].

The functionality of video cameras at these observatories in the context of multiparametric monitoring is of potential importance for bio-data production. One of the difficulties presently faced by marine research is the lack of sensors for the direct biological monitoring at the individual, population, and species levels. Most currently installed biological sensors measure life processes only indirectly, reporting habitat changes in terms of chemical derivates (e.g., dissolved oxygen, chlorophyll or nitrates) [21]. In contrast, geophysical and oceanographic sensors are more abundant and capable of directly measuring the processes of interest. Video imaging may fill this gap because the automated analyses of digital products enable the efficient counting of animals at a timing corresponding to other monitored habitat parameters [22].

In the last 30 years, a technological effort has been undertaken in the development of video imaging in association with multisensory measurements for habitat characterisation [1]. Video footage or frames acquired by means of Remote Operated Vehicles (ROVs), Autonomous Underwater Vehicles (AUVs), and nonpermanent camera stations have been used to portray with differential efficiency a broad range of processes such as bioturbation (*i.e.*, sediment structure disturbance), processes affecting marine snow, species behaviour and biotic interactions [23,24]. The characterization of biological processes could be performed with video cameras at cabled observatories by extending the video acquisition over large temporal windows (in association with multiparametric data collection). In this fashion, the integrated time series analyses of bio-data (*i.e.*, time series of visual counts for different species) and habitat parameters could help to establish causality in ecosystem functioning. These types of analyses are justified by the observation that animal physiology is affected by changes in habitat parameters such as salinity, temperature, pressure, current speed, and light intensity, and these physiological changes induce behavioural responses in terms of activity or passivity [25].

Herein, we describe the recently deployed cabled Seafloor Observatory (OBSEA), located within a protected coastal area in the western Mediterranean Sea. This multiparametric platform has various oceanographic sensors, including a video camera. The main goals of the observatory are to monitor the local coastal ecosystem at a relatively low cost and to provide an easily accessible infrastructure for the testing and development of new marine sensors. Accordingly, the hardware (the sensors and the network of marine and land nodes), software (data acquisition, transmission, processing, and storage), and long-term multiparametric data acquisition (habitat and bio-data time series) capabilities of the platform are described. An automated video image analysis protocol for counting fish in the water column was also developed. This method, which can be adapted for still cameras on coastal cabled observatories, couples bio-data time series with data from other oceanographic sensors to demonstrate the ability of the OBSEA platform to monitor coastal ecosystems remotely and continuously in real time. Accordingly, we firstly described the potential of OBSEA as a multiparametric measurement system of diversified habitat parameters and afterward, we presented an automated video imaging protocol used to produce biodata to be coupled within the biotope context. In doing so, our aim was to promote a discussion of the role of video camera systems as general sensors operating at the ecological complexity levels of animals (*i.e.*, by object tracking) and species (*i.e.*, by object classification).

## 2. System Architecture

**Figure 1.**
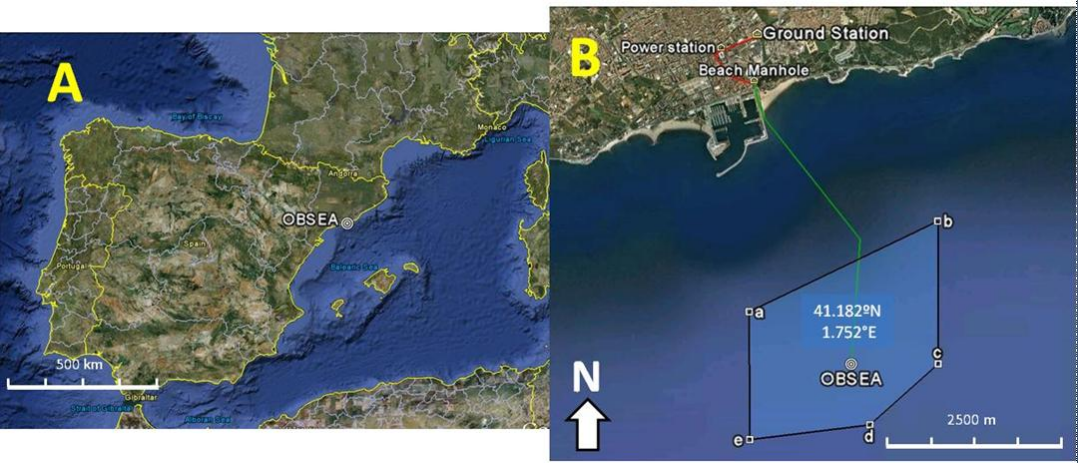
The location of the OBSEA seafloor cabled observatory in the western Mediterranean Sea **(A)**, with details of its localisation off the Catalan coast **(B)**. The cable routes in the sea and on the land (B) are indicated by green and red lines, respectively. OBSEA is located in a protected fishing area (*i.e.*, the “a–e” polygon shown in B).

The OBSEA was installed in May, 2009 by the R/V *Sarmiento de Gamboa* at a depth of 20 m in the marine reserve *Colls Miralpeix*, 4 km offshore of Vilanova i la Geltrú (Catalan Coast, western Mediterranean: 41°10′54.87″N and 1°45′8.43″E) (Figure 1). The installation of the platform was recorded in the scientific documentary “314” by the National Spanish Broadcasting Television System (RTEVE; http://www.rtve.es/alacarta/#755090; last accessed 10 February 2011). The OBSEA project [26] was conceived as a shallow-water infrastructure component of the EMSO-ESFRI Infrastructure (European Multidisciplinary Seafloor Observatory; Framework Program-FP7 Infrastructures-2007-1, Proposal 211816), and some activities were included in those of the Network of Excellence ESONET (European Seas Observatory NETwork; Framework Program-FP7 Infrastructures-2005-Global-4, ESONET 036851-2).

OBSEA is a technologically expandable platform. The Technological development Centre for Remote acquisition and data processing system (SARTI) completely developed OBSEA, integrating several commercial devices for power supplies and communications and designing all of the control systems and mechanical structures. In their first stages, some ideas and concepts were taken from the published documentation of other observatories, but all of the designs were done by the research group. The current system has only one node in the sea. In the future, other nodes will be placed at greater depths along and across the continental margin to create a network covering different areas relevant to commercial fisheries and conservation [27].

### 2.1. The Cable

The land station is connected to a submarine telecommunication cable (Figure 1) that stretches 1,000 m from land to the main sea node. The cable, which is composed of six single-mode optical fibres for data transmission, one central copper conductor tube, and one aluminium shielding sheet, enables continuous transmission of data and power. The power system is equipped with a cluster of AC/DC converters capable of producing up to 320 VDC and 11 A. The negative pole of the copper conductor is connected to the power supply, and the positive pole is connected to the aluminium cable shield and the ground.

### 2.2. The Junction Box and Sensors

**Figure 2.**
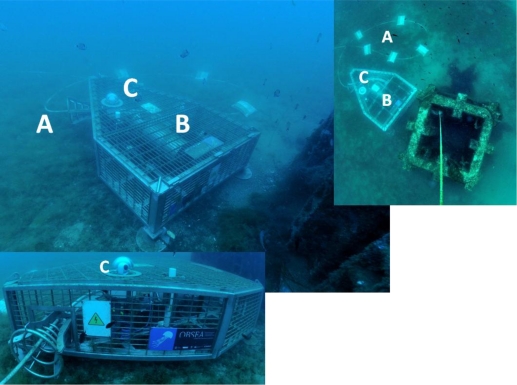
Oblique, vertical, and close lateral views of the OBSEA cabled seafloor observatory showing three structural elements: **(A)** the cable powering the platform instruments; **(B)** the junction box within the cylinder holding the installed sensors; and **(C)** the video imaging system. The concrete column used for studies on faunal colonisation is also visible close the observatory.

The sea node (Figure 2) is surrounded by a metallic cage to protect the instruments from unauthorised access. The cage contains the junction box within a cylinder, and the junction box contains a power supply, electronics for communication and control of the node, and connectors for the cable and all oceanographic instruments.

**Figure 3.**
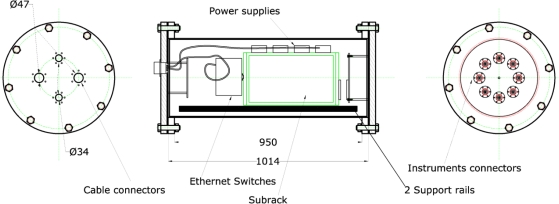
The architecture of the main cylinder (indicated by B in Figure 2) holding the junction box and the sensors within the OBSEA seafloor cabled observatory. Element sizes and distances are reported in millimetres.

The OBSEA marine node hosts several instruments including a video imaging system for bio-data acquisition. All of the available sensors are listed in Table 1. These sensors are connected to the node by cables that adapt their signals to the OBSEA Ethernet 10/100 interface (Figure 3). Two industrial Ethernet switches control communications between the marine node and the land station using two redundant 1 Gbps optical fibre links with a 1 + 1 configuration and TCP/IP protocols. These switches simultaneously relay signals from the sensors to the control system (Section 2.4).

The node distributes energy to all of the connected sensors and transmits acquired data to the shore station. It also controls the status of all connected elements through a control server (see Section 2.4). The power system consists of four emergency batteries and five switching converters, of which two are 300/48 V and three are 48/12 V. Voltages in the range of 80 to 370 VDC are accepted after passing through the redundant 1 + 1 150 W AC/DC converters.

### 2.3. The Control System of the Marine Node

The control system of the marine node relies on a 32-bit microcontroller (ColdFire MCF5282) to manage information flow via a Simple Network Management Protocol (SNMP). SNMP is a standard protocol of the TCP/IP family that allows system administrators to supervise the functioning of different elements within a network and to identify and solve potential failures [28]. This protocol consists of an agent and a manager. In OBSEA, the agent is the software that controls and monitors the functioning of the sea node. The manager is the software that is executed at the shore station and monitors the elements of the land network. With this configuration, the microcontroller can monitor and control the energy requirements and the state of connections of all of the sensors, thereby ensuring the correct functioning of the entire node. The system accepts input commands and generates alerts via an alternative console (RS232) that can be used for communication in emergencies.

To manage data acquisition from the sensors, two peripheral devices were included in the sea node: AD1232PROXR and XR16xDPDT (National Control). The former samples, quantifies, and converts incoming signals from the various sensors from analogue to digital format. The latter contains the relay drivers that control sensor functioning.

### 2.4. The Shore Station

The data management system at the land node stores time series of data from OBSEA sensors and makes these data accessible to web clients. A router provides internet access and enables access control and protection. The system has several servers for different tasks including oceanographic data management, SNMP network element supervision, and video storage. These servers are labelled as follows: *i. Lluna* connects low-bandwidth devices and stores data from all oceanographic sensors in a SQL database; *ii. Pop* stores video and uses the Zone Minder software for video processing; *iii. Medusa* uses the Zabbix software to mediate SNMP control of all network devices; and *iv. Server-OBSEA* provides Internet access and acts as a firewall in Linux. Additional servers are dedicated exclusively to the management of sensors that require supplementary data processing: *i. Lab* processes sounds from the hydrophone and *ii. Server-AWAC* processes Doppler data for current, wave and pressure calculations.

### 2.5. The Data Management System

The basic mechanism for data transmission and interactions among the sensors, the various system interfaces, and all potential OBSEA users was based on a model of overlapping service layers: *i.* the Instruments & Sensors Layer; *ii.* the Instruments & Sensors Interfaces Layer; *iii.* the Standard Formatting Layer; and *iv.* the Services Layer. In the System Architecture, instrumentation and sensors are located at the centre, and the next layer represents the available sensor interfaces using Ethernet protocols such as TCP and UPD. Data from the sensors is formatted using standards such as NMEA, SensorML or IEEE1451. The last layer is composed of a group of different services located around the Formatting Layer, in which the potential data clients and mechanisms for user applications are represented.

- Instruments & Sensors Layer: Represents the different measurement equipment and sensors deployed at OBSEA
- Instruments & Sensors Interfaces Layer: All instrumentation is connected to the OBSEA Local Area Network (LAN). Serial instruments use COTS (commercial off the shelf) serial-to-Ethernet converters, offering communication with the instrument using IP protocols such as TCP, UDP or SNMP.
- Standard Formatting Layer: Instrument information uses standard protocols for data interchange and transmission to the service layers consisting of ASCII datagrams in broadcast mode normalised over the standard NMEA-183 and the User Datagram Protocol (UDP), SensorML or HTTP IEEE1451.1
- Services Layers: In continuous development, OBSEA daily offers new Services for different clients in the Services Layers. Some of these are as follows:
  - Access to time series registers. Time series of all acquired data are saved independently, both on each platform and in the central node of the network. In the central node, a copy with 1-min synchronisation is also saved. This structure provides the redundancy required for safe data acquisition.
  - Raw and processed files. Raw data are stored in ASCII format with a NMEA datagram and in CSV format, with codified names and variable data. All ASCII files have daily extensions and are named with the date on which they were created and an extension indicating the instrument from which they were recorded.
  - Relational database service (SQL). SQL data are stored on three servers (Section 2.4): *i.* OBSEA, the primary server within the sea node; *ii.* MORFEO, which provides data access to the shore station; and *iii.* MEDUSA, which stores data on an SNMO server for alarm control. This layer stores time-specific data related to geographical extensions (POSTGRES+POSTGIS). Geographic measurement information is important because OBSEA will become a junction box, wherein instruments or platforms (both static and mobile) located in the same area will generate data through OBSEA. For this reason, each measurement must have a geographic reference.
  - Data services (WMS and WFS). These allow data to be geographically referenced on maps using the WMS and WFS standards of the Open Geospatial Consortium. Requests are initiated by users using the HTTP protocol as a communication channel between WMS and WFS servers.
  - Data export service (EXPORT). An export layer was constructed over the two outermost layers with the various interchange formats used in the marine context. The SensorML format has been proposed as a standard for data storage.
  - f Data service management (NMA). This layer allows for the synchronous transmission of data through TCP channels to the ZABBIX network manager. This service also allows for the monitoring of physical devices in the network and, thus, introduces the concept of data quality.
  - Data services (KMZ). This service provides the most recently acquired data in compressed KML format, with a real-time update of the contents and structure. This service is oriented to a Google Earth client.
  - Habitat data and their storage. LDAP trees have been proposed for mapping the network of sensors and instruments and the configuration and calibration files. The export layers of time series of data from SensorML or OpenDAP would read the information stored in these trees.

## 3. Multiparametric Time Series Acquisition

Multiparametric monitoring of oceanographic variables together with the platform’s remotely controlled digital camera provide a unique suite of instruments for interdisciplinary studies on individual and population behaviour as well as species presence in relation to cyclic and contingent habitat changes [11]. Habitat and bio-data are monitored to fulfil one of the goals of cabled observatory technology: the assessment of cause-and-effect relationships between temporal habitat changes and the observable (*i.e.*, counted) individuals of a species [10].

To demonstrate the utility of OBSEA in this context, data were acquired in a temporally limited (*i.e.*, one month) fashion in 2009 and 2010 with the available sensors (see Table 1). Data from the various sensors were not always acquired simultaneously. Acquisition months were different for CTD, ADCP, and digital images (see below) because OBSEA is a technological testing platform, and continuous data acquisition was not available year-round. Moreover, data from the hydrophone and seismometer were not used because these devices collected data at smaller time intervals than desired (e.g., hours or days), and long time series (e.g., weeks) were not available. All obtained data from the observatory were stored in a Zabbix management server to represent oceanographic data in parallel with observatory status data in order to identify possible periods of malfunction for quality control.

### 3.1. The Measurement of Oceanographic Parameters

The main goal of the OBSEA platform is to provide a relatively low-cost, continuous, remote and long-term monitoring of the local coastal ecosystem in terms of physical and biological parameters. The scientific objective of this observatory is to increase our knowledge of the very low-frequency processes occurring on the wave-dominated inner shelf by means of long-term monitoring. In the future, we expect to be able to identify and quantify changes in environmental parameters and to provide tools for the analysis of climate-change impacts and the improvement of the integrated management of the coastal area.

The real-time acquisition of oceanographic data by each sensor was tracked with screen interfaces, which were either provided by the manufacturer or developed with a special software program called Data Turbine, which is a streaming server and manages data acquisition from several sensors. Time series data were collected for approximately one month with the CTD sensors (1–29 June 2009) to measure conductivity, temperature, and pressure and with the ADCP sensor (24 March–22April 2010) to measure current speed and direction, turbidity, and chlorophyll concentration. The ADCP was deployed on a bottom tripod in an upward-facing configuration and collected information in water cells spaced 1 m apart. Waves were measured hourly during bursts of 8.5 minutes at 2 Hz, and currents, chlorophyll and turbidity were measured every 10 minutes by averaging 1-minute burst measurements at 1 Hz. The turbidity and chlorophyll sensors were placed 0.5 m above the seafloor.

At present, wave and current data are only checked by the transformation software provided by NORTEK. In relation to the control of CTD data quality, there are two alarms configured on the Zabbix server software, which verify if data are being received and which is their value in relation to the trend. In addition, the alarm system also controls the power consumption of sensors and detects possible malfunctions. CTD data are manually controlled once per week (including the acquisition periods under description here). Also, CTD temperature is cross-checked with the temperature recorded on divers’ computer every month during observatory maintenance operations (e.g., video camera cleaning).

**Figure 4.**
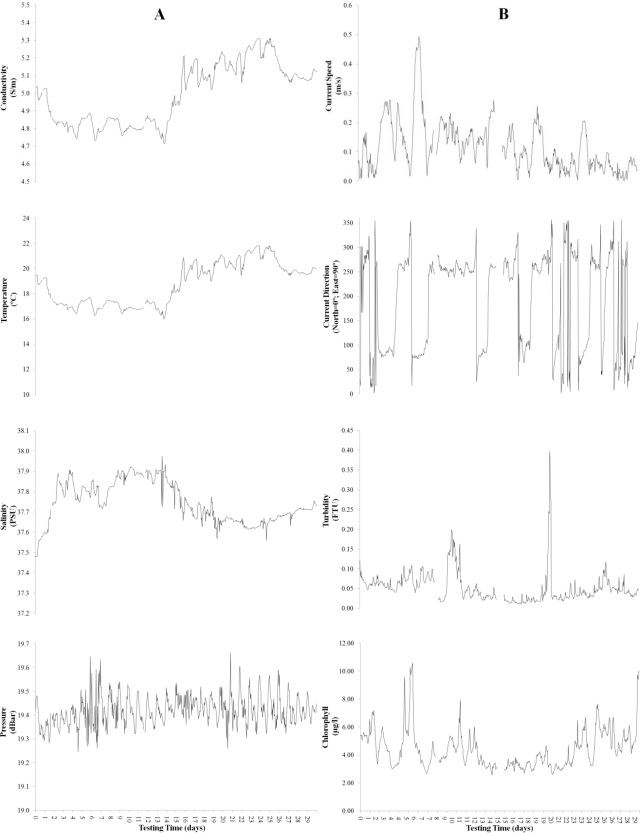
One-month time series of processed hourly oceanographic data, as recorded by CTD **(A)** and ADCP **(B)** sensors installed on the OBSEA seafloor cabled observatory (see Table 1 for references on their specifications).

Time series data for CTD (conductivity, temperature, and pressure) and ADCP (current speed and direction, turbidity and chlorophyll concentration) were downloaded and preprocessed to obtain average estimates at a frequency of 1 hour. The data series are shown in Figure 4. Moments of sensor malfunctioning produced blank spaces within time series.

### 3.2. Video Imaging Measurements

The OBSEA video imaging system was used to capture temporal fluctuations in the populations of fish in the local community. The objective was to demonstrate the efficiency of video cameras as sensors for the production of bio-data, which can be coupled with other habitat data to obtain an integrated analysis of temporal ecosystem functioning. Video image bio-data were gathered from 5 September to 30 November 2009; this period is different from the previously detailed oceanographic parameters due to camera unavailability at that time.

**Figure 5.**
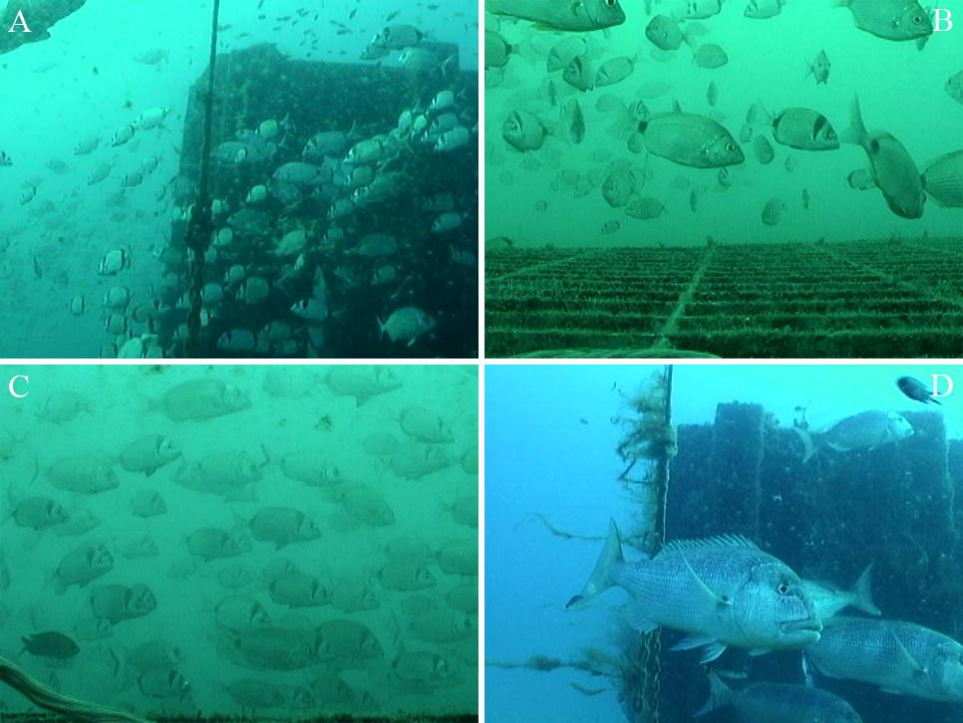
Fish species commonly detected in the OBSEA seafloor cabled observatory area by the installed video imaging system (indicated by C in Figure 2). Given the capability of the imaging system to rotate on its axis by 360°, different views of the water column and the concrete column for colonisations studies are presented. **(A)** *Diplodus vulgaris* and *D. annularis*; **(B)** *Diplodus vulgaris* and *D. annularis*; **(C)** *D. sargus*, *D. annularis*, and *Chromis chromis*; **(D)** *C. chromis* and *Dentex dentex*.

To illustrate the difficulties associated with automated video image analysis in coastal environments, an example of the fish diversity observed by the OBSEA video imaging system is shown in Figure 5. Commonly observed fish species [29] include the two-banded seabream (*Diplodus vulgaris*), the damselfish (*Chromis chromis*), the black seabream (*Spondyliosoma cantharus*), the white seabream (*Diplodus sargus*), the annular seabream (*Diplodus annularis*), and the common dentex (*Dentex dentex*).

Images in jpg format at a resolution of 480 × 360 pixels (72 dpi) were manually acquired every 60 min during daylight hours from the same position (B in Figure 5). Images were not taken at night because an illumination system had not been installed. Frames were manually acquired in the absence of a suitable automated protocol.

**Figure 6.**
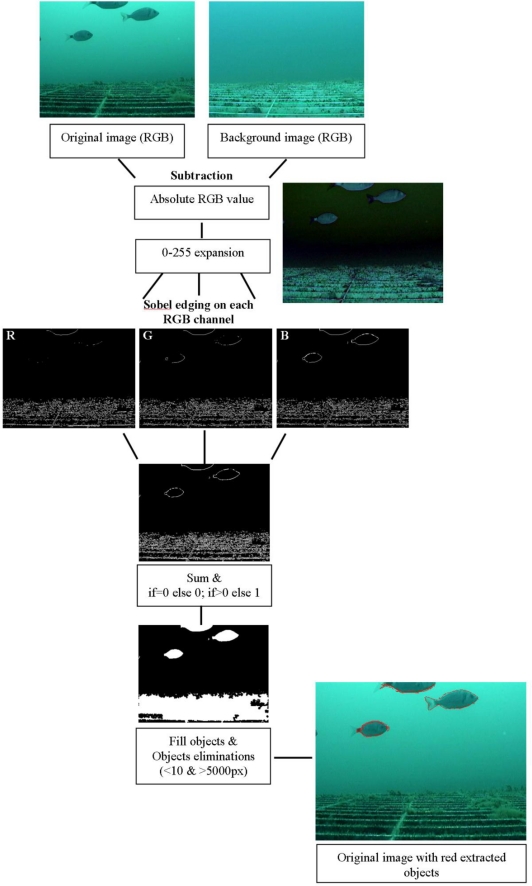
Diagram of the processing steps in the automated video image analysis protocol used to count fish in digital frames acquired by the OBSEA seafloor cabled observatory.

A total of 804 images were processed with a customised script in MATLAB 7.1, the steps of which are shown in Figure 6. A general reference image without fish was used for subtraction with all other processed images (*i.e.*, image subtraction). For each resulting image, the absolute values of the pixels were considered. Each image was rescaled in the interval from 0 to 255 (*i.e.*, expansion). Sobel edging was performed on each R, G and B channel [30,31] to obtain three respective binary images. Each single pixel at the same position in each image was then summed following these criteria: if a pixel value was 0, then the null value was preserved, and if a pixel value was greater than 0, then the value was changed to 1. The resulting images were again rescaled from 0 to 255. White objects were then filled. Objects with pixel sizes within the interval of 10 to 5,000 were considered; all others with smaller and larger values eliminated. Finally, the number and cumulative area of objects in each image were extracted.

**Figure 7.**
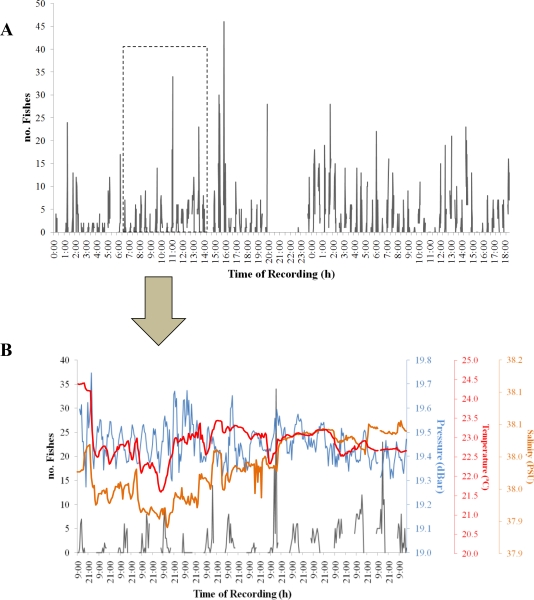
Time series of visual fish counts (black line) as produced by the automated video imaging analysis of still frames acquired by the OBSEA seafloor cabled observatory video imaging system. **(A)** The whole three-month time series of fish visual fish observations. **(B)** A 15-day subset (from 18 September to 3 October 2009) is presented (black line) together with corresponding CTD measurements (for a period not included in the previous oceanographic measurements). The latter comparison was performed as an example of potential interdisciplinary multiparametric coupled habitat and bio-data acquisition.

This procedure produced visual counts of all fish, but it did not make species distinctions (Figure 7). Thus, the method successfully estimated animal count fluctuations within a group at the taxonomical ranking of Superorder (*i.e.*, Teleostei). A time series subset was also compared with data derived from the CTD sensors to produce a graphical output example of coupled multiparametric habitat and bio-data acquisition. In our case, habitat and bio-data time series coupling can reveal how fluctuations in fish counts can be related to variations in water parameters as the product of the animals’ perceived changes in the ecofield [25].

Difficulties in the classification of animals at the species level were caused by the varying distances between animals and the camera, by postural changes, and by the clustering of animals (*i.e.*, schools) in the camera region of interest (ROI). Clustering affected the general performance of automated individual counting. Frames also showed different lighting conditions, and animal motion within the water column prevented sizing by automated analysis because metric tools, such as background scale references, lasers [32], dual-camera systems [33,34] and structured lighting [35,36], had not been installed on the platform. In an important step, a reference set of images was created [22] to calculate the different types of errors in the processing procedure. Of the 804 images, 500 (62.5%) were manually processed for fish counts, and the outputs were compared with those from the automated analysis. It is a common procedure to choose a subset of images for manual *versus* automatic comparisons [22]. The following counting errors were identified:

1. Images (Img) that did not contain fish were automatically classified (Class) as containing fish (*i.e.*, Img0-ClassN)
2. Images that contained fish were automatically classified as not containing fish (*i.e.*, ImgN-Class0)
3. Images in which the number of manually counted fish (<20) was greater than the number of automatically counted fish (*i.e.*, ImgN-ClassM). We considered a manually counted number of fish larger than 20 to be a school
4. Images in which the number of manually counted fish was greater than 20 but the number of automatically counted fish was less than 10 (*i.e.*, Img > 20-Class < 10)
5. Images in which the number of manually counted fish was less than 20 but the number of automatically counted fish was greater than 10 (*i.e.*, Img < 10-Class > 20)

**Figure 8.**
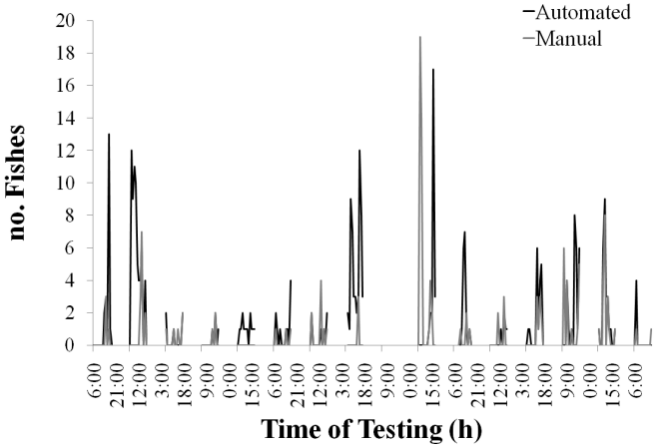
A two-week graphical comparison (from 8–23 September 2009) of the time series obtained by manual counting (*i.e.*, visual inspection) and automatic fish identification from images taken by the camera of the OBSEA seafloor cabled observatory. This manual *versus* automatic time series comparison shows the efficiency of the elaborated automated processing protocol.

**Figure 9.**
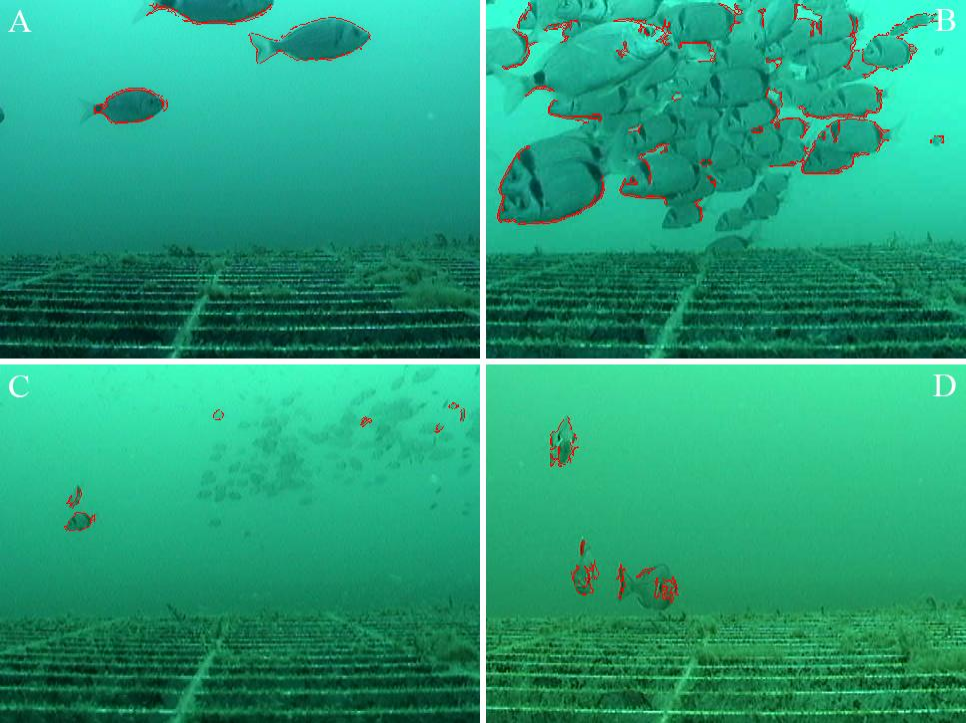
Examples of different types of errors (see Table 2 for references on their typologies) encountered during the automated processing of images taken by the OBSEA cabled seafloor observatory video camera: **(A)** is an example of an image with correctly classified fishes; **(B)** is an example of an image with a fish school (>20 animals) classified as containing less than 10 individuals (Img > 20-Class < 10 in Table 2); **(C)** is an example of an image in which a distant school is not fully differentiated from the background; and **(D)** is an example of an image where the resampling of the same object occurred (*i.e.*, M > N in Table 2).

A time series comparison of automatic and manual fish counts for a representative subset of images (177 images) is shown in Figure 8. In Table 2, the results of the statistics on the error estimations are presented in detail. Among the 500 images of the test set, 264 (52.8%) contained fish according to manual counts. Conversely, automated counts indicated that a total of 314 images (62.8%) contained fish. Approximately 31.6% of the images (66.9% of the images without objects) were correctly classified as not containing fish (Correct0), whereas manual and automatic counts yielded the same number of fish in 18.4% of images (34.8% of the images with fish) (CorrectN; see below in Figure 9(A)).

According to the error typologies detailed in Table 2, Figure 9 illustrates different visual examples of automatic counting mismatches. The results of the error analysis are as follows: 15.6% of the images did not contain fish but were classified as containing a mean of 2.9 animals (Img0-ClassN); 4.4% of the images contained a mean number of 4.3 fish but were classified as not containing fish (ImgN-Class0); 11.6% of the images contained more than 20 fish but were classified as containing less than 10 (Img > 20-Class < 10) (fish in dense schools were difficult to count (Figure 9(B)) or were difficult to differentiate from the background (Figure 9(C)); no images containing less than 10 fish were classified as containing more than 20 (Img < 10-Class > 20); 18.4% of the images contained a different number of fish (ImgN-ClassM), and when M > N (*i.e.*, 52 times), this was mainly caused by resampling of the same object (*i.e.*, 29 times; Figure 9(D)).

Based on this error analysis, an overall evaluation of the automated video image analysis protocol was conducted. Approximately 50% of the images in the test set were automatically analysed without fish counting errors. Approximately 10.4% had small errors; the difference between the manual and automatic counts was approximately threefold. With the automated protocol, 20% of the images in the error classes Img0-ClassN and ImgN-Class0 had low average values for incorrect counting (*i.e.*, 2.9 and 4.3, respectively).

## 4. Discussion

Herein, we described the hardware, software, and data acquisition capabilities of the OBSEA cabled marine observatory, and the preliminary results were presented. Long-term (*i.e.*, weeks) time series data for different oceanographic parameters and visual fish counts were shown to demonstrate the scientific value of this platform for remote and continuous real-time monitoring in coastal ecosystems.

Long-term data sets are increasingly required to study ecosystem functioning in relation to human-induced environmental changes, which are occurring over progressively larger geographic areas [37]. Any discussion of long-term habitat changes caused by human actions, for example, variations in the presence and abundance of certain species as indicators (e.g., Lessepsian migrations), should rely on long-term data. This procedural need is captured in the expression “the longer, the better” for habitat and bio-data acquisition [1]. These observations justify the creation of new devices for remote, continuous and automated data acquisition.

In this context, the main objective of OBSEA is to provide a test bed for the technological modification, adaptation, and implementation of oceanographic instrumentation and the simultaneous production of valuable information for the scientific community. Currently, all long-run temporal marine observations are stored in a database at the shore station, with the exception of video camera data, the automation of which is still under development. All real-time data are accessible to the scientific community through the Internet, and stored data can be obtained on request. The importance of social and scientific feedback [38] emphasises the importance of providing the scientific community with access to large data sets *via* the Internet (http://www.obsea.es). Virtual collaboration among groups of researchers from different institutions or different geographic areas is fostered through the sharing of analyses and conclusions in a common web-based workspace [39]. Network sensors enable real-time access to measurements and the interactive control of remote assets [40]. An optical Ethernet network manages real-time sensor data acquisition and status monitoring (using an SNMP protocol).

In oceanographic observation, high resolution, large volumes of information and long data series are becoming increasingly important because traditional observation systems (e.g., autonomous buoys and measurements taken from vessels) present serious disadvantages regarding the costs, volume and delayed transmission of data, and face limitations of battery autonomy [40]. The newer cabled underwater observatories, in contrast, are modular, flexible and adaptable to different uses and specifications. Cabled observatories allow the interdisciplinary long-term monitoring of the oceans, combining data acquisition from different fields in an effort to understand ecosystem dynamics at different scales [41] with the use of various underwater oceanographic sensors [42,43]. Additionally, with the use of video imaging systems, important time series of bio-data related to temporal variations in species presence or population size can be also obtained [44].

The adaptation of video technology for the monitoring of behaviour in the field is the first step in the study of population behavioural rhythms that are at the root of biases in biodiversity and stock assessments [45,46]. The results presented here indicate two important areas of difficulty in the use of this technology: animal counting and classification. In the proposed protocol, only the former is addressed; fish counts were performed, but species classification was not. We reported errors that could not be remedied by the parallel use of other, more traditional techniques such as acoustics (echo sounders) that were not available on the OBSEA platform. Imaging errors reported in the automated counting were likely due to the following: *i.* markedly different background illumination at different hours of the day, which was also contingent on climatic conditions (e.g., cloudiness), affected counts; *ii.* the absence of metric references obscured measurements of the depth field of the ROI; *iii.* the schooling behaviour of coastal fish obscured counts; and *iv.* species outlines were similar. Based on these sources of error, we propose the use of a uniform marine-blue background shield located 4–5 m from the camera field to create a homogeneous background and the use of a colour chart within the ROI to facilitate the automatic discrimination of species by the colour (RGB ratios) of their automatically detected shapes (*i.e.*, most local fish species have similar shapes, and only colorimetric analysis can be used to classify them correctly).

Among visual census experts, the general opinion is that direct diving is the best method for estimating biomass [47]. Results from the video image analysis suggest the importance of remote monitoring with cabled observatories in the management of marine resources compared with other, more traditional sampling techniques such as visual censuses. The advantages of remote monitoring include the high frequency of sampling over large time periods (from months to years) and the ability to avoid the effects of divers on fish behaviour. However, disadvantages are also associated with this method. Contextual factors such as turbidity, cloudiness, brightness, and camera fouling, which can add additional noise, affect frame and data quality. The results indicate that the video acquisition of still images is a reliable technique that can be automated for species recognition and animal tracking. With more processed images, consistent long-term studies based on comprehensive biological data can be conducted.

It should be noted that OBSEA currently represents a single observational point, but it will be expanded in the near future to greater depths on the continental margins and horizontally at similar water depths. If cabled observatories are punctual windows of observation, then these local tools are an efficient source of data for ecological modelling related to species' responses to deterministic habitat changes (*i.e.*, geophysical cycles in light intensity and tides) [7]. The expansion of video observatories to larger portions of the continental margin areas (*i.e.*, by means of networks) will help to define the presence of fish species and diel (either diurnal or nocturnal) movement across depth gradients [48].

OBSEA can be used for long-term monitoring because it can be continually updated to address the challenges associated with the complexity of marine data measurement. Human societies have primarily developed along coasts, which are now experiencing significant anthropogenic perturbations [49]. As a result, various environments are increasingly threatened, including ecosystems at continental shelf margins and slopes and in the deep sea [50]. In this context, adding on-shore meteorological and hydrological observations may be of help in assessing the effects of meteorology on coastal marine processes. OBSEA data can be retrieved and correlated with information from external sources, such as climatic ground observatories located near the shore station (real-time data is available at http://www.meteoclimatic.com/perfil/ESCAT0800000008800B), to obtain advanced conclusions on the responses of sea communities to climate variations. The scientific community and other organisations (e.g., governmental funding agencies) are increasingly interested in extensively monitoring the marine environment to solve current and future problems related to the sustainable use and management of marine resources [51]; for this purpose, technology has been developed for the continuous and automated measurement of habitat and biological data [52]. Based on its ability to collect habitat and bio-data, OBSEA can fulfil the needs of long-term studies related to the conservation and renewable management of marine coastal resources.

## 5. Conclusions

The newly installed cabled coastal observatory, OBSEA, was described in terms of its functioning. A real-time observation of multiple parameters in the marine environment was efficiently achieved by means of a platform providing continuous power to sensors and having a high-bandwidth communication link. This multiparametric platform was used to acquire data on several oceanographic parameters related to fish bio-data for use as outputs from the automated analysis of digital products acquired with the installed video imaging system. In this sense, we attempted not only to describe the potential of this new cabled observatory but also to propose a new methodological approach based on the use of video cameras in an integrated and parallel fashion with the other oceanographic sensors. Within this framework, we developed an automated video image analysis protocol suitable for working in a water column space, with consideration of all difficulties related to that compartment of the marine environment. Independently of this protocol, and given sufficient time, video analysis automation will likely increase in efficiency, forming a basis for the establishment of sound cause-and-effect relationships between temporal community variations and habitat fluctuations simultaneously measured with oceanographic sensors at a corresponding periodicity.

## Figures and Tables

**Table 1.** Types and technical characteristics of sensors installed on the OBSEA seafloor cabled observatory.

CTD	SeaBird SBE-37SMP
	Conductivity range: 0–7 S/m (acc. 0.0003, res. 0.00001)
	Temperature range: −5–35 °C (acc. 0.002, res. 0.0001)
	Pressure range: 0–35 atm (acc. 0.35, res. 0.007)
	Sampling interval: 10 s
	Derived parameters: salinity, depth, and sound propagation

ADCP	Nortek AWAC (Acoustic Wave and Current meter) with Surface
	Tracking
	4 transducers of 1 MHz acoustic beams with compass and tilt sensors for correction of the deployment position
	Range: 10 m/s (1% of measure accuracy ±0.5 cm/s)
	Derived parameters: current speed and direction and surface wave height, period, and propagation direction.
	Integrated additional sensors: for turbidity meter (Seapoint; max sens. 200 mV/FTU, range 0–25 FTU) and fluorimeter (for chlorophyll concentration determination; (Cyclops; sens. 0.025 μg/L, range 0–500 μg/L).

TV-camera	Ocean Presence Technologies OPT-06 Underwater IP Camera (Sony SNC-RZ25N)
	Minimum resolution: 640 × 480 (Mpeg/Mjpeg); 18 × optical zoom
	Minimum light sensitivity: 0.7 lux
	Measuring purposes: animal tracking and species classification

**Table 2.** Number and percentage of correct (Correct0 and CorrectN) and incorrect fish counts (others) for the automated video image analysis protocol compared to manual counts. Treated images were obtained by the video camera installed on the OBSEA seafloor cabled observatory.

**Correct0**	158 (31.6%)
**CorrectN**	92 (18.4%)
**Img0-ClassN**	78 (15.6%); mean: 2.9 objects
**ImgN-Class0**	22 (4.4%); mean: 4.3 objects
**ImgN-ClassM**	92 (18.4%); mean (M > N): 2.9 objects; mean (M < N): 3.7 objects
**Img>20-Class<10**	58 (11.6%)
**Img<10-Class>20**	0 (0%)
